# New insights in chemokine signaling

**DOI:** 10.12688/f1000research.13130.1

**Published:** 2018-01-23

**Authors:** Daniel F. Legler, Marcus Thelen

**Affiliations:** 1 Biotechnology Institute Thurgau (BITg), University of Konstanz, Kreuzlingen, Switzerland; 2 Institute for Research in Biomedicine, Università della Svizzera italiana, Bellinzona, Switzerland

**Keywords:** Chemokine, chemokine receptor, signal transduction, GPCR

## Abstract

Chemokine signaling is essential for coordinated cell migration in health and disease to specifically govern cell positioning in space and time. Typically, chemokines signal through heptahelical, G protein-coupled receptors to orchestrate cell migration. Notably, chemokine receptors are highly dynamic structures and signaling efficiency largely depends on the discrete contact with the ligand. Promiscuity of both chemokines and chemokine receptors, combined with biased signaling and allosteric modulation of receptor activation, guarantees a tightly controlled recruitment and positioning of individual cells within the local environment at a given time. Here, we discuss recent insights in understanding chemokine gradient formation by atypical chemokine receptors and how typical chemokine receptors can transmit distinct signals to translate guidance cues into coordinated cell locomotion in space and time.

## Chemokine-induced signaling

Soon after chemokines were discovered in the late ’80s ^1– 3^, it was shown that their cognate receptors on cell surfaces were members of the rhodopsin-like family of G protein–coupled receptors (GPCRs). The sensitivity of intracellular signaling to pertussis toxin indicated that the putative receptor for the orphan ligand CXCL8 (formerly interleukin-8[IL-8]) expressed on human neutrophils couples to the G _i_ class of heterotrimeric proteins ^4^. A few years later, the receptors CXCR1 and CXCR2 were identified, cloned, and expressed on mammalian cells for studying signaling properties ^5, 6^. Despite their high sequence identity (almost 80%), ligand selectivity is different for the receptors. CXCL8, CXCL5, and CXCL6 bind to both CXCR1 and CXCR2, but the latter also binds the chemokines CXCL1-3 and CXCL7 ^7^ with high affinity. Hence, chemokines can bind multiple receptors, and on the other side receptors are not always selective for one specific chemokine. Moreover, CXCR1 and CXCR2 differ in their capacity to induce cellular responses upon stimulation with CXCL8. Both receptors stimulate intracellular calcium fluxes, chemotaxis, and degranulation; however, only CXCR1 stimulation leads to activation of phospholipase D and the respiratory burst in human neutrophils ^7, 8^. These early observations not only indicated a promiscuity within the chemokine system but also revealed that the GPCRs have the ability to couple differently to downstream signaling pathways. Moreover, a given chemokine can stimulate different responses depending on the receptor to which it binds as well as on the cells where the receptors are expressed.

Typically, chemokine receptor stimulation leads to the GDP/GTP exchange of coupled heterotrimeric G _i_ proteins and the subsequent dissociation of the βγ subunits, which then activate phosphoinositide-specific phospholipase Cβ (PLC) and phosphoinositide 3-kinase (PI3K). PLC produces inositol-trisphosphate (IP _3_) and diacylglycerol (DAG). IP _3_ triggers calcium mobilization whereas DAG activates protein kinase C (PKC). PI3K generates 3-phosphoinositides, which serve as anchors in the recruitment of proteins with pleckstrin homology domains to the plasma membrane, such as AKT/PKB ^9^. Although these signaling events are common to all chemokine receptors, it is well known that the activation of further downstream pathways is quite different. This may depend on the efficacy with which a chemokine triggers its receptor, giving rise to different spatial and temporal signal fluxes. Such biased signaling at the chemokine receptor was recently revealed by using biosensors to demonstrate differences in G-protein subclass coupling of CCR2, CCR5, and CCR7 ^10^. Other important considerations are the surface expression and density of a receptor and the specific cellular context. As an example, the second ligand of CCR7 CCL19, in contrast to CCL21, does not attract T cells in a microfluidic migration assay under flow conditions ^11^ but does efficiently stimulate migration of cells transfected with the receptor ^12, 13^, dendritic cells ^14, 15^, or T cells in static migration assays ^16^. Moreover, monocyte-derived dendritic cells express CCR7 on the cell surface but migrate toward CCL19 and CCL21 only when matured in the presence of prostaglandin E _2_^17, 18^. A recent observation indicates that GPCRs move within restricted areas of the cell surface. These membrane subdomains are maintained by “fences” created by the cytoskeleton and “pickets” made of transmembrane proteins. At special hot spots, GPCRs and G proteins are retained and preferentially couple ^19, 20^. These findings imply that signaling by GPCRs can be confined on the cell surface and may depend on the local availability of downstream interaction partners.

## Signal bias

Several observations indicate that chemokine receptors may not exclusively couple and signal through G proteins but interact with additional signaling mediators, such as β-arrestins. Upon activation, GPCRs become desensitized through phosphorylation of their intracellular C-termini by second messenger–dependent kinases and GPCR kinases (GRKs). The phosphorylation pattern, also known as barcode, induces arrestin recruitment to the receptor ^21, 22^. However, depending on the ligand-mediated stimulation, the recruited β-arrestins cause receptor inactivation and internalization or the receptor-bound arrestin acts as a scaffold which leads to the recruitment and activation of protein kinases ^23– 27^. An early definition suggested that agonists which induce receptor internalization are considered G protein–biased but that those which trigger arrestin-dependent signaling are called β-arrestin–biased ^22^. A more complete view of biased signaling takes into account that signal bias can depend on the ligand (ligand bias), the receptor (receptor bias), and the context (tissue bias) ^28, 29^. For CXCR4, it was shown that monomeric and dimeric forms of CXCL12, which both may exist under physiological conditions ^30^, induce selective signal transduction pathways and differ in β-arrestin recruitment ^26, 27^. Whereas dimeric CXCL12 does not induce β-arrestin recruitment and chemotaxis, both monomeric and dimeric forms of CXCL12 equally trigger the activation of ERK ^26^. For CCR7, CCL19 binding results in robust serine/threonine phosphorylation of the receptor and β-arrestin recruitment catalyzed by GRK3 and GRK6, whereas CCL21 binding activates GRK6 alone ^31^. Consequently, CCL19 induces rapid CCR7 internalization whereas CCL21 hardly does ^32^ and hence can be seen as ligand bias ^15^. Notably, GRK6 contributes to haptotactic sensing of CCL21 gradients at least by dendritic cells ^33^.

Ogilvie *et al*. showed that CCR2 can activate distinct cellular responses depending on the chemokine which binds to the receptor ^34^. Also, this observation can be seen as ligand bias; however, it does not depend on receptor phosphorylation. Whereas CCL2 induces all typical responses when used to stimulate CCR2, such as calcium fluxes, actin polymerization, and chemotaxis, CCL11 instead was shown in binding assays to act as an antagonist and to suppress CCL2-induced signaling ^35^. More detailed analysis revealed that CCL11 triggered pertussis toxin-sensitive ERK phosphorylation downstream of CCR2 without inducing GDP/GTP exchange of the G protein or leading to receptor phosphorylation. Activation of ERK was required to antagonize CCL2-mediated signaling by CCR2. Both chemokines stimulated PI3K; however, CCL2 stimulated the βγ-dependent PI3Kγ isoform whereas CCL11 activated a p85/p110 isoform ^34^. In general, ligand binding to GPCRs induces the rearrangement of the transmembrane helices ^36^. The above observations are consistent with a view where CCL2 and CCL11 induce different conformations of CCR2, which translate to diverse intracellular coupling.

Binding of CXCL10 to CXCR3 drives T helper 1 (Th1) polarization via STAT1, 4, and 5 phosphorylation, whereas CXCL11 induces a Th2 and regulatory T (Treg) (IL-10 ^hi^) fate involving p70 kinase/mTOR and STAT 3 and 6 ^37^. The marked differences in T-cell polarization could be explained by the chemokine-specific signaling. In an early study, which did not investigate T-cell fate, it was shown that the three ligands of CXCR3 (namely CXCL9, CXCL10, and CXCL11) induce typical responses such as calcium mobilization and chemotaxis. By contrast, upon stimulation, CXCR3 internalization was most prominent with CXCL11 whereas CXCL9 and CXCL10 showed only moderate effects. The differences were explained with the use of distinct entities of the intracellular domains of CXCR3 to transmit the responses when stimulated with CXCL9 and CXCL10 versus CXCL11 ^38^. More recently, it was shown that CXCL11 and, to a lesser extent, CXCL10, but not CXCL9, induce β-arrestin2 recruitment ^39^. Interestingly, more pronounced differences were reported for β-arrestin recruitment and the binding modality to the two splice variants CXCR3A and CXCR3B, which differ by a 51–amino acid extension at the extracellular N-terminus of CXCR3B ^25, 39^. However, expression of the putative CXCR3B in mouse tissue is not clear and this is due to an in-frame stop codon in the coding exon ^40^. These observations confirm that intracellular coupling efficiency of the receptor can be modulated by extracellular ligand binding.

## Modulation of chemokine receptor signaling

Chemokine activity on cognate receptors can be modulated in multiple ways. The nuclear protein HMGB1, which is released by necrotic or severely stressed cells, binds TLR4 and RAGE but not chemokine receptors. However, HMGB1 forms heterocomplexes with CXCL12, which stimulate CXCR4 with higher potency than the chemokine alone ^41^. Moreover, chemokines can act synergistically, increasing their potency and efficacy of receptor activation ^42– 44^. In addition, chemokine receptors, when triggered with two chemokines, can display allosteric regulation. For example, CXCL14 binds CXCR4 with high affinity but does not stimulate any typical receptor-mediated response. Nevertheless, CXCL14 markedly enhances the potency and efficacy of CXCL12 on CXCR4 ^45^.

Direct interaction of chemokine receptors with G proteins, GRKs, and β-arrestin is amply reported. In addition, second-messenger kinases, such as PKC and PKA, phosphorylate serine and threonine residues at the C-termini of chemokine receptors. However, some chemokine receptors were shown to directly bind and activate additional proteins, giving rise to receptor-specific activation of signal transduction. CXCR4 interacts with the eukaryotic translation initiation factor eIF2B, suggesting that the receptor may stimulate local protein synthesis ^46^. Indeed, mesenchymal cells were shown to *de novo* synthesize actin in the cell periphery ^47, 48^. CCR7, when oligomerized, is able to bind and activate an Src kinase signaling pathway which leads to tyrosine phosphorylation within its DRY motif, which then serves as a docking site for SH2 domain–containing molecules such as phosphatase SHP2 ^49^. Similarly, the kinase JAK2 was shown to phosphorylate CCR2B upon stimulation with CCL2 ^50^. Another direct interaction is the binding of VASP to CXCR2 necessary to mediate CXCL8-stimulated cell migration ^51^. The interaction of FROUNT with CCR2 and CCR5 enhances migration of monocytes and macrophages by increasing consolidated pseudopodium formation ^52, 53^.

## Chemokine presentation

The chemokine system is well known to orchestrate leukocyte migration through the formation of chemotactic gradients. It should be noted that such chemotactic gradients are locally confined, not exceeding 100–150 µm ^54^. Local confinement implies that chemokines are retained on cell surfaces and the extracellular matrix ^55^. Glycosaminoglycan (GAG) binding sites can be found in all chemokines and were shown to be essential to mediate the binding to proteoglycans. Binding of chemokines to GAGs can modify their activities, enhancing or reducing their potency on cognate receptors ^55, 56^. On the other side, GAG binding can increase local chemokine concentrations (for example, in receptor vicinity) and efficiently present the ligands for haptotacic chemokine receptor-mediated migration of cells. Secondary B-cell follicles are characterized by germinal centers (GCs) where B-cell antibody affinity maturation occurs. The GCs are split into the CXCL12-rich dark zone, where B-cell centroblasts proliferate, and the CXCL13-rich light zone, where centrocytes are selected for antigen affinity ^57^. Specific stroma cells, the CXCL12-expressing reticulate cells (CRCs), produce CXCL12 in the dark zone ^58^, whereas follicular dendritic cells release CXCL13 in the light zone ^59^. During affinity maturation, B cells move between the two compartments of the GC, being attracted reciprocally by the two chemokines ^57^. In transgenic animals which express CXCL12 lacking GAG binding sites, the dark zone is enlarged and poorly defined, consistent with the notion that CXCL12 needs to be locally retained to maintain the structure of the GC, which is not surrounded by physical borders ^60^. Similarly, CXCL13 can bind to GAGs without losing its capability to bind to CXCR5, being able to promote adhesion-dependent cell migration ^61^. However, additional mechanisms, which attenuate B-cell migration at the periphery of GCs, were shown to be essential for efficient B maturation and GC integrity ^62^.

## Atypical chemokine receptors

An important consideration for the generation and maintenance of biological gradients was made by Francis Crick, who proposed that, in apposition to a source of a morphogen, a sink must exist in order to prevent the gradient from blurring ^63^. Cells migrating on chemokine gradients scavenge the ligands from the surrounding medium and in this way presumably contribute to gradient maintenance ^64^. In addition, the group of atypical chemokine receptors (ACKRs), which share the seven-transmembrane domain topology of conventional chemokine receptors but do not couple to G proteins and fail to induce typical intracellular signaling, act as scavengers targeting chemokines for lysosomal degradation ^65, 66^. ACKR4 (formerly CCRL1), a scavenger of the chemokines CCL19, CCL21, and CCL25, is expressed on the lymphatic endothelium (LECs) of subcapsular sinuses (SCSs) of lymph nodes. In the SCSs, the expression of ACKR4 is asymmetric, being present on LECs forming the ceiling of the SCSs but not on those on the floor facing the interfollicular areas. The asymmetric distribution generates CCL21 gradients pointing from the SCS across the floor LECs into the interfollicular areas ^67^. This CCL21 gradient is assumed to be critical for dendritic cell and T-cell emigration from SCSs into the parenchyma of lymph nodes. For ACKR3, a scavenger for the chemokines CXCL11 and CXCL12 ^68^, it was shown, in zebrafish lateral line primordium as a model, that the migrating cell collectives can self-generate CXCL12 gradients across their length ^69, 70^. In humans, ACKR3 is upregulated on B cells at the plasmablast stage, when cells downregulate CXCR5 and exit the GCs ^71^. Because ACKR3 has about a 10-fold higher affinity for CXCL12 than CXCR4, it was concluded that expression of the scavenger renders the cells less sensitive to CXC12-mediated retention via CXCR4 in the GCs allowing egress. Indeed, migration of plasmablast toward CXCL12 is markedly reduced but can be rescued upon attenuation of ACKR3 ^71^.

Signaling through the chemokine system not only plays a role in hematopoietic cells but also is present in mesenchymal cells. Chemokine signaling is required during development in the central nervous system ^72– 74^. ACKR3 was shown to be critical for the migration of interneurons in mouse brain development. The role of the scavenger appears to lie in the control of the level of CXCL12. In the absence of the scavenger, excess of CXCL12 leads to the downregulation of CXCR4 which causes the attenuation of interneuron migration ^75, 76^. The chemokine system also plays a pivotal role in angiogenesis, where chemokines induce cell growth and stimulate the recruitment of endothelial cells ^66^. The properties of the chemokine system have been adopted by many neoplasms. Several lines of evidence indicate that metastatic infiltration of distant organs such as the bone marrow, lung, and liver is mediated by chemokines and their cognate receptors ^77– 79^. In a recent study ^80^, the infiltrating properties of human diffuse large B-cell lymphomas (DLBCLs) into distant organs in a disseminated mouse xenograft model were tested. While organ infiltration is assumed to depend on CXCR4-mediated migration, expression of ACKR3 appeared to play a critical role. In the absence of the scavenger, the DLBCLs fail to infiltrate the organs. *In vitro* studies suggest that ACKR3 is required to generate local CXCL12 gradients during extravasation ^80^.

## Conclusions

Although all typical chemokine receptors expressed on leukocyte are able to induce cell migration, the signaling mechanisms downstream of the receptors are not unified. Rather, a complex signaling network composed of biased signaling, promiscuous signaling, and signal specificity paired with chemokine presentation and scavenging contributes to chemokine-stimulated cell migration. Such fine tuning is important to allow specific and efficient migration (for example, during immune responses) to guarantee precise spatiotemporal localization of individual effector cells.
